# *Et tu, Brute*? TFEB promotes virus replication before being cleaved by a viral protease

**DOI:** 10.1080/27694127.2024.2402675

**Published:** 2024-09-16

**Authors:** Alagie Jassey, William T. Jackson

**Affiliations:** Department of Microbiology and Immunology and Center for Pathogen Research, University of Maryland School of Medicine, Baltimore, USA

**Keywords:** TFEB, virus, autophagy, nonlytic release, NEAR

## Abstract

The relationship between many viruses and cellular autophagy is complex; autophagy is a useful mechanism of membrane rearrangement and trafficking, but autophagosomes can degrade virus components and newly formed virions. Picornaviruses, whose relationship to autophagy has been studied for almost thirty years, cause a range of diseases, from mild respiratory illnesses such as the common cold to major paralysis syndromes such as poliomyelitis and acute flaccid myelitis. Like most viruses with RNA genomes, picornaviruses translate, replicate, and package their genome in the cytosol, often in association with membranes. Early in picornavirus infections, there is an extensive rearrangement of intracellular membranes to form a replication platform, the cytosolic surface of which serves as the site for viral RNA replication. Later in infection, autophagosome-like vesicles bud from the replication organelle and are used to traffic viruses to the cell surface, releasing virus packets surrounded by the former inner membrane of the double-membrane autophagosome-like vesicles. Thus, picornaviruses must inhibit the degradative aspects of the pathway to prevent viral proteins or even newly formed virions from being consumed in autolysosomes. With this in mind, we recently described the dual-natured role of host TFEB (transcription factor EB), in the picornavirus virus cycle. TFEB is required for early infection, but cleaved by a viral protease late in infection as part of a viral strategy to subvert the autophagy machinery to remodel membranes while inhibiting degradative autophagy. This is a story out of Shakespeare, in which viruses betray a cellular ally in a manner worthy of Brutus; first usurping the function of TFEB, then cleaving that same protein when the needs of the virus change late in infection.

Although autophagosomes are robustly induced late in picornavirus infection, they do not mature into degradative autolysosomes because picornaviruses can employ multiple strategies to block autophagosome-lysosome fusion. The most common strategy is to cleave proteins essential for autophagic flux, including the tether PLEKHM1 (pleckstrin homology domain-containing protein family member 1) and SNAP29 (synaptosome-associated protein 29), which both regulate autophagosome-lysosome fusion. This blockade of autophagosome fusion benefits picornaviruses by preventing the degradation of virions or their components, and facilitating RNA replication, capsid maturation, and virion egress from cells.

TFEB, a member of the microphthalmia family of basic helix–loop–helix–leucine–zipper (bHLH-Zip) transcription factors, is the master transcriptional regulator of autophagy and lysosomal biogenesis. The nuclear/cytoplasmic localization of TFEB depends on both its phosphorylation/dephosphorylation by mechanistic target of rapamycin, extracellular signal-regulated kinase, and calcineurin and interaction with the Rag GTPases. Previous work from the Luo laboratory showed that TFEB is cleaved during Coxsackievirus B3 (CVB3) infection by the viral 3C protease, impairing lysosomal function and promoting CVB3 nonlytic release. This finding indicates that TFEB cleavage could be an additional mechanism to block autophagic flux during CVB3 infection by obstructing lysosomal acidification. Thus, we decided to investigate a role for TFEB during infection of enterovirus D68 (EV-D68), a related picornavirus and primary causative agent of the childhood paralysis disease acute flaccid myelitis.

In our recent publication, we showed that knockdown of TFEB reduced EV-D68 intracellular titers in both wild-type H1HeLa and ATG7-KO H1HeLa cells.^1^ Knockdown of TFEB impaired viral RNA levels without exerting any significant effect on viral binding and entry into host cells. However, immediately after the peak of viral RNA replication, the 3C protease of EV-D68 cleaved TFEB at the N-terminus. A similar cleavage was carried out by the CVB3 3C protease, and we have observed a similar TFEB cleavage in Poliovirus-infected cells, indicating that multiple picornaviruses target this host factor. Thus, TFEB is useful to the virus early in infection but cleaved late in infection.

The cleavage disrupts the TFEB-RagC interaction, which is essential for the cytosolic retention of TFEB. This is in agreement with the original CVB3 data, in which the newly cleaved (and inactive) TFEB translocates to the nucleus late during infection. However, despite cleaving TFEB at an identical site, TFEB remains largely cytosolic during EV-D68 infection. Overexpression of a TFEB mutant construct lacking the RagC binding domain blocked autophagic flux and enhanced EV-D68 nonlytic release. This effect was abrogated in autophagy-deficient cells lacking the *ATG7* gene[1].

In the early stages of infection, TFEB promotes EV-D68 RNA replication, between 0 h to 3 h post infection. The transcription function of TFEB is not essential in promoting viral RNA replication. Blocking general transcription with actinomycin-D did not affect viral intracellular titers. We believe TFEB either directly promotes the formation of viral RNA replication organelles or recruits necessary host proteins to viral RNA replication sites. The early function of TFEB does not depend on autophagy. However, in the later stages of infection, TFEB is cleaved, resulting in inhibition of autophagic flux, facilitating the autophagosome-mediated nonlytic release of EV-D68. Our data indicate that EV-D68 fine-tunes TFEB function to benefit multiple stages of the virus cycle, using both autophagy-dependent and autophagy-independent mechanisms.

If the localization of TFEB differs among picornaviruses, then why is TFEB cleavage important for these viruses? We suggest that TFEB cleavage may be an additional mechanism by which picornaviruses block autophagic flux. The cleavage products generated during viral infection may act as dominant negatives, impairing the transcriptional activity of TFEB in different ways for each virus, such as blocking TFEB nuclear import for EV-D68, or attenuating TFEB binding to promoters for CVB3. This suggests that autophagic flux can be obstructed either by TFEB mislocalization or TFEB dysfunction. There is also another possibility. We have recently uncovered that inducing autophagic flux via acute amino acid starvation immediately upon EV-D68 infection abrogates EV-D68 replication. Recent unpublished data from our lab shows that several autophagy regulatory proteins from the initiation, nucleation, and elongation complexes are localized to the nucleus. These autophagic regulators evacuate the nucleus during acute amino acid starvation to drive stress-induced autophagy, something also shown to occur with LC3 protein. While LC3 nuclear evacuation is regulated by acetylation, the nuclear evacuation of the other autophagy proteins is not regulated by acetylation. Instead, they are regulated by TFEB in a process we call Nuclear Evacuation of Autophagic Regulators (NEAR). Knockdown of TFEB attenuates NEAR and impairs the antiviral activity of stress-induced autophagy. We hypothesize that picornaviruses cleave TFEB to prevent NEAR and avoid stress-induced autophagy during picornavirus infection (Figure 1).

**Figure 1.**
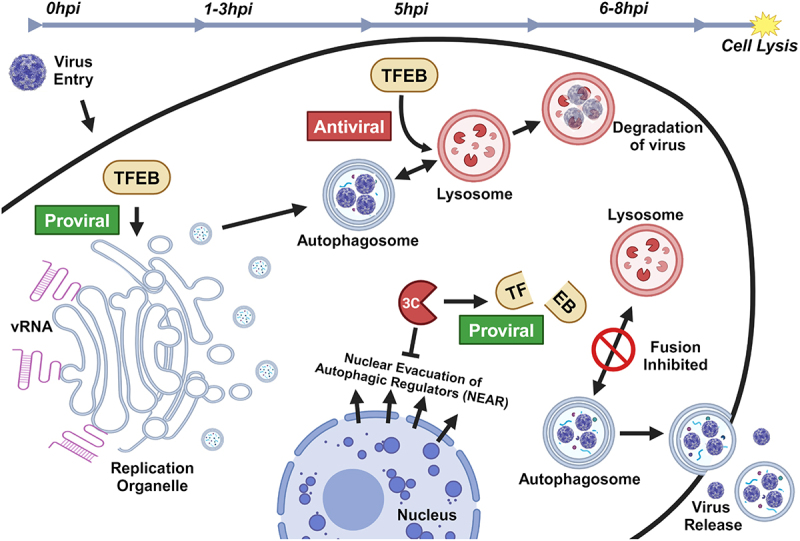
Proviral and antiviral roles of TFEB during EV-D68 infection.

TFEB cleavage, like an assassination on the Ides of March, seems like a brutal way to treat a protein the virus needed just a few hours earlier. Reminiscent of the betrayal of Caesar, our findings about TFEB during EV-D68 infection capture the duality of the relationship between picornaviruses and autophagy. TFEB’s function is critical for the membrane rearrangements induced by the virus, but once that function has been served, TFEB must be cleaved to avoid the action of antiviral, degradative autophagy while at the same time promoting secretory autophagy. It is a niche in which picornaviruses have it both ways; usurping autophagic membranes while blocking autophagic degradation.

## Abbreviations

EV-D68: Enterovirus D68; CVB3: Coxsackievirus B3; ATG7: autophagy related 7; TFEB: transcription factor EB; PLEKHM1: pleckstrin homology domain-containing protein family member 1; SNAP29: synaptosome-associated protein 29; bHLH-Zip: basic helix–loop–helix–leucine–zipper; NEAR: Nuclear Evacuation of Autophagic Regulators.

## References

[1] Jassey A, Pollack N, Wagner MA, Wu J, Benton A, Jackson WT. Transcription factor EB (TFEB) interaction with RagC is disrupted during enterovirus D68 infection. J Virol 2024; 98:e0055624. 10.1128/jvi.00556-24 PMC11265353 38888347

